# Understanding Nurses’ Experiences of Fragmented Care in Aging Populations: A Meta-Synthesis

**DOI:** 10.1097/jnr.0000000000000720

**Published:** 2026-01-09

**Authors:** Cheng CHENG, Martin CHRISTENSEN

**Affiliations:** 1 School of Nursing, Fudan University, Xuhui, Shanghai, China; 2 School of Nursing, Hong Kong Polytechnic University, Hung Hom, Kowloon, Hong Kong SAR, China

**Keywords:** aging, fragmented care, meta-synthesis, nurses, qualitative research

## Abstract

**Background:**

Despite global population aging, fragmented care remains a significant obstacle to the provision of effective health care, particularly to older adults with complex health needs.

**Purpose:**

This study was designed to synthesize current qualitative research on nurses’ experiences with care fragmentation in caring for aging populations.

**Methods:**

A comprehensive search of two electronic databases was conducted to identify relevant literature published in English. A total of 20 articles covering a total of 296 nurse participants were included in this synthesis. The extracted data were thematically synthesized.

**Results:**

Four main themes and 10 subthemes were identified in the synthesis. The themes included (1) challenges in accessing and delivering health care services, (2) challenges in communications and coordination, (3) the need to advance nurses’ roles and recognize their contributions, and (4) the importance of providing patient-centred care.

**Conclusions/Implications for Practice:**

The findings of this study highlight the complexities and challenges of providing care to aging populations within fragmented health care systems. The difficulties faced by nurses in accessing resources, coupled with communication and coordination barriers, underscore the need for systemic improvements to facilitate effective care delivery. The theme related to nurse recognition highlights the need for greater support and acknowledgement of the contributions of nurses, which is essential to ensuring care quality and patient outcomes. Moreover, the emphasis on patient-centered care reinforces the importance of adopting individualized care strategies that cater to the unique needs of older adults. Health care systems should prioritize initiatives aimed at improving communication and coordination among health care providers, supporting nursing staff, and implementing patient-centered care practices. These efforts are crucial to overcoming the challenges of fragmented care and ensuring older populations receive the comprehensive and effective care they deserve.

## Introduction

The aging population is a rising global health concern, and the demand for health care services for older adults is growing quickly. Fragmented care is a common problem in health care systems, particularly in the care of older adults with complex health needs (Eastman et al., 2022).

The concept of fragmented care refers to the lack of communication and coordination among health care providers, settings, and services (Nothelle et al., 2022). Several factors previously identified as contributing to fragmented care in older adults include lack of a coordinated care model, poor communication and information sharing between health care providers, and the complexity of older adult health needs (Kaneko et al., 2022; Mathew & Patel, 2021). Moreover, fragmented care has been shown to negatively impact patient outcomes in terms of higher rates of hospitalization and emergency department visits and decreased quality of life (Prior et al., 2023). Also, fragmented care may increase overall health care costs, as service duplication and conflicting treatments may result in unnecessary tests and procedures (Gogna et al., 2024).

In several studies, systemic changes, including improved communication and collaboration between health care providers, increased resources and support for nurses, and a shift toward patient-centered care, have been proposed to address the issues related to fragmented care (Barnea et al., 2021; Sumpter et al., 2022). However, implementing these changes will require a multifaceted approach involving health care providers, administrators, policymakers, and patients. Nurses are uniquely positioned to identify and address fragmented care due to their holistic approach to care and their frequent interactions with patients and families (Karam et al., 2021).

Various settings, including acute care hospitals or long-term care facilities, have been considered in the existing literature on nurses’ experiences of fragmented care. Qualitative findings indicated that physicians, nurses, and unlicensed assistive personnel (UAPs) often function in parallel rather than as an integrated team, with minimal interpersonal communication (Lancaster et al., 2015). Also, nurses face difficulties in coordinating care, communicating with other health care providers, and accessing necessary resources (Östman et al., 2021). Furthermore, previous studies have identified role confusion, ethical dilemmas, and other challenges faced by nurses in delivering care in fragmented systems. The disparate research on this issue indicates the need to synthesize findings and identify common themes and patterns across different settings and populations and the need to identify best practices and strategies for addressing fragmented care from a nursing perspective. The meta-synthesis approach facilitates the synthesization and interpretation of qualitative findings from individual studies to provide a deeper understanding of the complex issues surrounding fragmented care (Dawson, 2019).

This study was designed to address the abovementioned gaps in current scholarly knowledge using a meta-synthesis of the existing literature on nurses’ experiences of fragmented care in aging populations. The authors expect the findings of this study to contribute to the development of best practices and strategies for addressing fragmented care in the context of aging and to inform the design of more coordinated and patient-centered care delivery systems, which will be critical to improving patient outcomes and reducing health care costs.

## Methods

In meta-syntheses, data from multiple qualitative studies are combined and analyzed to offer new insights and understandings (Lachal et al., 2017). The conduct of the meta-synthesis in this study followed the systematic review process outlined in MacMillan et al. (2019). All reporting was conducted in accordance with the enhancing transparency in reporting the synthesis of qualitative research (ENTREQ) statement (Tong et al., 2012). This meta-synthesis was registered on the OSF platform and is located at osf.io/5yv69.

### Research Question

The research question for this meta-synthesis was “What are the experiences and perceptions of nurses regarding fragmented care when caring for older adults?” This question was developed using the Population, Phenomenon of Interest, and Context (PICo) framework (Lockwood et al., 2015).

### Literature Search

A comprehensive search of the literature was conducted using the CINAHL and MEDLINE electronic databases for related reports published in English at any time. The search terms used were free words and MeSH terms, as determined by the application of each database, including “fragmented care,” “coordinated care,” “nurses’ experiences,” “older adults,” “ageing populations,” and “qualitative research.”

### Study Selection

The inclusion criteria for this meta-synthesis were studies (1) focused on nurses’ experiences with fragmented care, (2) focused on the health care provided to older adults, (3) that used a qualitative research design, and (4) that were published in a peer-reviewed English language journal. The exclusion criteria were studies (1) focused on the experiences of non-nursing health care professionals (e.g., physicians, surgeons, nursing students), (2) that were not primary studies, or (3) not published in English. Mixed-methods studies were also excluded.

A comprehensive literature search across the two databases conducted in March and June 2024 yielded a total of 852 records. All of the 852 articles were retrieved and evaluated for relevance using the EndNote X9 reference tool. Two of the authors first screened the titles and abstracts independently and then reviewed the full-text versions of the retained articles. Any disagreements were resolved using group discussion, and a third researcher was available for consultation if needed.

**Figure 1:**
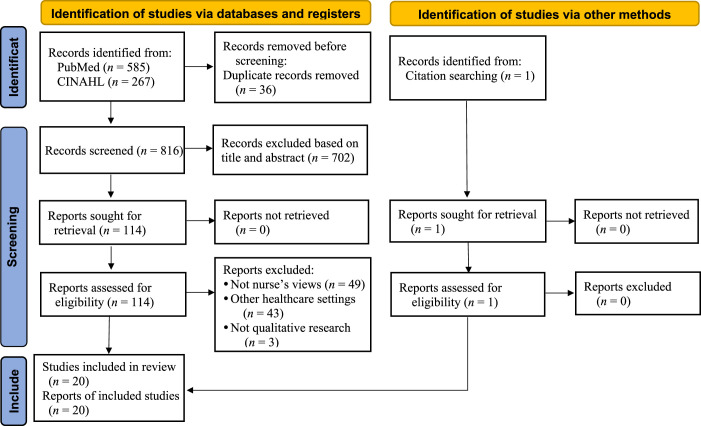
Preferred Reporting Items for Systematic Reviews and Meta-Analyses (PRISMA) Flowchart

After removing 36 duplicates, a two-step screening process was used to further refine the selection. The initial screening of titles and abstracts resulted in 114 articles meeting the inclusion criteria, while the subsequent full-text review narrowed the selection down to 19 articles deemed relevant for inclusion in the synthesis. One additional article was identified through other sources and included in the final selection, bringing the total number of articles included in the analysis to 20. The selection process used in this study is depicted in the Preferred Reporting Items for Systematic Reviews and Meta-Analyses (PRISMA) flowchart in Figure 1.

### Quality Appraisal

The quality of the included studies was appraised by two independent reviewers experienced in qualitative research with expertise in the field of gerontology. The quality of the included studies was evaluated using the Critical Appraisal Skills Programme 2023 tool for qualitative research, which consists of 10 questions designed to assess the rigor and credibility of the study, the relevance of the study to the research question, and the depth and richness of the data. Any discrepancies in quality ratings were discussed and resolved through consensus. As the objective was to enhance the overall rigor of the meta-synthesis, no study was excluded based solely on the basis of quality. The results of the quality appraisal are presented in Table 1.

**Table 1:** Quality Appraisal of the Included Studies (*N*=20)

Article ^a^	Q1	Q2	Q3	Q4	Q5	Q6	Q7	Q8	Q9	Q10
Magilvy & Congdon (2000)	Y	Y	Y	N	Y	Y	N	U	Y	Y
Hughes & Goldie (2009)	Y	Y	Y	Y	Y	N	Y	Y	Y	U
Huber et al. (2011)	Y	Y	Y	Y	Y	N	Y	Y	Y	Y
Ljungbeck & Sjögren Forss (2017)	Y	Y	Y	Y	Y	N	Y	Y	Y	Y
Mc Namara et al. (2017)	Y	Y	Y	Y	Y	N	Y	Y	Y	Y
McInnes et al. (2017)	Y	Y	Y	Y	Y	N	Y	Y	Y	Y
A. I. I. King et al. (2018)	Y	Y	Y	Y	Y	N	Y	Y	Y	Y
Borglin et al. (2019)	Y	Y	Y	Y	Y	N	Y	Y	Y	Y
Dowson et al. (2020)	Y	Y	Y	Y	Y	Y	Y	Y	Y	Y
Freilich et al. (2020)	Y	Y	Y	N	Y	N	Y	Y	Y	Y
Haase et al. (2020)	Y	Y	Y	Y	Y	N	Y	Y	Y	Y
Irani et al. (2020)	Y	Y	Y	Y	Y	N	Y	Y	Y	Y
Peart et al. (2020)	Y	Y	Y	Y	Y	Y	Y	Y	Y	Y
Dolu et al. (2021)	Y	Y	Y	Y	Y	Y	Y	Y	Y	Y
Persson et al. (2022)	Y	Y	Y	N	Y	U	Y	Y	Y	Y
Santos Oliveira et al. (2022)	Y	Y	Y	Y	Y	N	Y	Y	Y	Y
Agerholm et al. (2023)	Y	Y	Y	Y	Y	U	Y	Y	Y	Y
Lindberg et al. (2023)	Y	Y	Y	Y	Y	N	Y	Y	Y	Y
Winqvist et al. (2023)	Y	Y	Y	Y	Y	N	Y	Y	Y	Y
Peeler et al. (2024)	Y	Y	Y	Y	Y	Y	Y	Y	Y	Y

*Note.* Q=question; Y=yes; N=no; U=unsure. Q1: Was there a clear statement of the aims of the research? Q2: Is a qualitative methodology appropriate? Q3: Was the research design appropriate to address the aims of the research? Q4: Was the recruitment strategy appropriate to address the aims of the research? Q5: Was the data collected in a way that addressed the research issue? Q6: Has the relationship between the researcher and participants been adequately considered? Q7: Have ethical issues been taken into consideration? Q8: Was the data analysis sufficiently rigorous? Q9: Is there a clear statement of findings? Q10: How valuable is the research?

^a^ Studies are listed chronologically.

### Data Extraction

Data were extracted from the included articles using a predeveloped Microsoft Excel form with the following information: (1) study characteristics (author[s], year of publication, country, study design, and methods), (2) participant characteristics (age, gender, profession, and years of experience), and (3) key findings.

### Data Synthesis

The data synthesis utilized an inductive thematic analysis approach well-suited for integrating and synthesizing qualitative findings from multiple studies (Braun & Clarke, 2006). This approach facilitates a nuanced understanding of the phenomenon by identifying recurring themes and patterns across diverse research contexts. The synthesis in this study followed a rigorous six-phase process (Maguire & Delahunt, 2017):

1. Familiarization with the data set: Researchers immerse themselves in the qualitative data from each study to understand their unique contexts and perspectives thoroughly.
2. Generate initial codes: Significant features of the data relevant to the research questions are captured in initial coding that is then used to identify emerging themes.
3. Search for patterns and connections: Systematic analysis of the initial codes helps uncover patterns and connections within and across studies, revealing shared experiences and insights.
4. Test potential themes: Potential themes are examined against the data to ensure they accurately represent the narratives and complexities. This iterative process refines the themes and accounts for variations in interpretations across studies.
5. Define and name themes: Each theme is clearly defined and named to encapsulate its essence and significance, ensuring clarity and coherence for effective communication of the findings.
6. Write a coherent report: The final report provides an integrated narrative of the identified themes.

## Results

### Summary of Included Reports

The 20 articles that met the inclusion criteria were published between 2000 and 2024, with six from Sweden (Agerholm et al., 2023; Borglin et al., 2019; Freilich et al., 2020; Lindberg et al., 2023; Ljungbeck & Sjögren Forss, 2017; Winqvist et al., 2023), four from Australia (Dowson et al., 2020; Mc Namara et al., 2017; McInnes et al., 2017; Peart et al., 2020), three from the United States (Irani et al., 2020; Magilvy & Congdon, 2000; Peeler et al., 2024), one from Ireland (Hughes & Goldie, 2009), one from Switzerland (Huber et al., 2011), one from New Zealand (A. I. I. King et al., 2018), one from Canada (Haase et al., 2020), one from Turkey (Dolu et al., 2021), one from Denmark (Persson et al., 2022), and one from Brazil (Santos Oliveira et al., 2022). The included studies included a total of 296 nurses, with the number in each ranging from 3 to 76. Demographic data on the participants were reported in most of the studies (Borglin et al., 2019; Dolu et al., 2021; Dowson et al., 2020; Freilich et al., 2020; Hughes & Goldie, 2009; Irani et al., 2020; Lindberg et al., 2023; McInnes et al., 2017; Peart et al., 2020; Persson et al., 2022; Santos Oliveira et al., 2022; Winqvist et al., 2023).

The included studies addressed a diverse range of health care settings, ranging from rural to urban areas and encompassed various care facilities, including hospitals, nursing homes, aged-care homes, and specialized centers (e.g., cancer hospitals). Purposive sampling was employed in most of the studies, while a smaller number used other techniques such as strategic selection, convenience sampling, and mixed sampling. Data were collected via interviews in all of the studies using either semi-structured or focus group formats. Additional sources of data such as field notes were employed in some of the studies. A range of techniques were employed to analyze the data, with content and thematic analyses the most frequently used approaches (each technique was applied, respectively, in six of the studies). The characteristics of the included studies are presented in Table 2.

**Table 2:** Characteristics of Included Studies ^a^

Author, Year/Country	Aim	Sampling	Data Collection and Data Analysis	Setting	Participant	Finding
1. Magilvy & Congdon (2000)/United States	To examine health care transition experiences of older adults, families, and health care professionals.	Unclear	• Structured interviews and data in forms of field notes, photographs, and articles; • Ethnographic analysis	Rural community health care center	76 nurses, demographic data unclear.	This study found that a cultural issue identified through analysis was the crisis state of health care transitions faced by rural older adults and their families, as observed by rural nurses and other health care professionals
2. Hughes & Goldie (2009)/Ireland	To examine adherence to medication and resident involvement in prescribing and decision-making of medicines.	Purposive	• Focus group interviews; • Constant comparison	Nursing home	9 female nurses, working experiences ranged from 3 to 15 years.	This study found that an aspect of health care transitions for rural older adults is the control of prescribing and administration processes, as well as the factors that affect control, and the balance between maintaining resident autonomy and ensuring control
3. Huber et al. (2011)/Switzerland	To examine nurses’ experiences in diabetes care for older adults in home health care and nursing homes.	Purposive	• Focus group interviews and field notes; • Thematic content analysis	Nursing home	23 nurses, demographic data unclear.	This study found the following issues related to diabetes care: (1) the current situation of diabetes care is marked by concerns about complications, interprofessional dependency, communication, and continuity, (2) nurses have reported apprehension regarding the varied interest in diabetes, the suitability of assessments, and their current knowledge of the disease and its impact on patient outcomes, (3) barriers to diabetes care, and (4) resources for diabetes care.
4. Ljungbeck & Sjögren Forss (2017)/Sweden	To examine the experiences of managers, doctors and nurses in primary care and municipal health care about the role of advanced nursing practitioners for frail older adults.	Strategic selection	• Semi-structured interviews; • Content analysis	Primary and municipal health care	4 nurses, demographic data unclear.	This study found that the role of the advanced nurse practitioner is multifaceted, as it addresses various perspectives, including (1) meeting the health care needs of the frail elderly, (2) enhancing the overall effectiveness of health care, (3) overcoming recruitment challenges, (4) improving the skills of municipal nurses, (5) fostering closer collaboration between municipal health care and primary care, and (6) inspiring nurses to pursue further education and take on the responsibilities of the role
5. Mc Namara et al. (2017)/Australia	To examine methods for multimorbidity management, and perceived barriers and enablers to deliver medication management for patients with multimorbidity and polypharmacy based on health care professional perspectives.	Purposive	• Semi-structured interview; • Framework approach	Urban and rural primary health care	6 nurses, demographic data unclear.	This study found that six factors in providing effective health care, including (1) the integration of shared decision-making and patient preferences, (2) the use of evidence-based practice, (3) the consideration of patient prognosis, (4) the clinical feasibility of treatment plans, (5) the optimization of therapies and health management plans, and (6) the coordination of care.
6. McInnes et al. (2017)/Australia	To examine the experiences of collaboration between nurses and general practitioners.	Purposive	• Semi-structured interview; • Thematic analysis	Urban and rural primary health care	14 female nurses, aged from 30 to 59 years old.	This study found that examining the general practice registered nurse’s (GPRN) role and its three subthemes is essential: (1) the importance of role clarity, (2) the GPRNs’ perception of their own identity, and (3) appreciating the GPRNs’ expertise.
7. A. I. I. King et al. (2018)/New Zealand	To explore the gerontology nurse’s role based on the perspectives of older people and health professionals.	Purposive	• Semi-structured interview; • General inductive approach	Primary health care	3 nurses, demographic data unclear.	This study found two major themes related to health care provision: proficiency and delivery of service.
8. Borglin et al. (2019)/Sweden	To examine nurses’ experience of identifying and intervening with patients with depressive symptoms.	Convenience	• Interviews; • Inductive content analysis	Primary health care	10 female nurses, working experiences ranged from 1 to 4 years.	This study found four overarching themes, which included (1) difficulties in identifying specific experiences, (2) the nature of interventions described, (3) necessary conditions for identification, and (4) the impact of external factors on participants’ experiences.
9. Dowson et al. (2020)/Australia	To examine the experiences of health professionals on antimicrobial use near the end of life and investigate the potential for nurses to perform antimicrobial stewardship activities.	Purposive, convenience and snowball sampling	• Semi-structured interviews and field notes; • Open and descriptive approach and integrated process of inductive and deductive coding	Aged-care home	12 female nurses, working experiences ranged from 4 months to 40 years.	This study found that after decisions to cease active treatment, careful antimicrobial use is pursued, though errors in its application were noted. Nurses were identified as key influencers in antimicrobial decisions in end-of-life care in aged-care homes, with the study commenting on the environmental and social contexts that shape nurses’ influence.
10. Freilich et al. (2020)/Sweden	To explore professionals’, patients’, and family caregivers’ experiences regarding support for self-management in patients with multimorbidity.	Unclear	• Interviews; • Inductive content analysis	Urban primary health care	3 nurses, aged from 37 to 60 years old, with working experiences ranging from 6 to 20 years.	This study found that establishing a mutual understanding facilitates personalized support. This central theme is underpinned by four key categories: (1) the importance of tailoring support to the individual and the role of patient-professional relationships in this process, (2) the function of professionals in translating knowledge to assist patients in acquiring self-management skills, (3) the challenge of managing and coordinating care for patients with multiple chronic conditions within a health care system primarily designed to treat single diseases, and (4) the evolving roles and varied perspectives regarding who is responsible for self-management.
11. Haase et al. (2020)/Canada	To examine experiences of using the Internet for health information and its role in the patient-provider relationship from both patients’ views and health care professionals’ views.	Purposive and theoretical	• Interviews and focus groups; • Thematic analysis	University-affiliated cancer center	12 female nurses, demographic data unclear.	This study found three overarching themes: (1) independently improving health care services and support mechanisms, (2) providing support through and properly contextualizing information, and (3) activating family and support networks to play a more significant role in the patient’s care.
12. Irani et al. (2020)/United States	To examine the factors of care planning for home care.	Convenience	• Semi-structured interviews; • Content analysis	Urban home health care center	20 nurses (18 females), mean age=46 years old, diverse racial identities, mean working experiences=9 years.	This study found three-factor themes: (1) social support, (2) the influence of the home environment and neighborhood, and (3) the challenges related to finances and insurance barriers.
13. Peart et al. (2020)/Australia	To explore the experience of providing care coordination to people living with multimorbidity based on health care professionals’ views.	Purposive	• Semi-structured interviews; • Interpretative phenomenological analysis	Hospital avoidance risk program teaching hospitals and several community health care services	11 nurses, working experiences ranged from 5 to 35 years.	This study found four overarching themes: (1) the difficulty in maintaining a focus on the individual patient, (2) the importance of “Hearing their story,” which involves actively listening and allowing clients the time to share their experiences, (3) the development of effective strategies for encouraging patient participation in the program, and (4) the necessity to “see the bigger picture.”
14. Dolu et al. (2021)/Turkey	To examine health care professionals’ experiences of transitional care from hospital to home.	Purposive	• In-depth semi-structured interviews; • Thematic analysis	Urban public and private community health care center	8 nurses (6 females), mean age=38.7 years old, mean working experience=22.5 years.	This study found three overarching themes: (1) an uninterrupted chain of care transfer to ensure seamless transitions between care settings, (2) a commitment to meet patients’ needs, which underscores the dedication required from health care providers, and (3) support and removing ambiguities to provide clarity and assistance throughout the patient’s care journey.
15. Persson et al. (2022)/Denmark	To examine health care professionals’ experiences regarding care coordination across sectors.	Unclear	• Semi-structured, interviews; • Systematic text condensation	Primary health and secondary health care center	8 nurses, work experiences ranged from 10 months to 28 years.	This study found four overarching themes: (1) organizational factors, (2) approaches to care, (3) communication and knowledge, and (4) relations.
16. Santos Oliveira et al. (2022)/Brazil	To examine nurses’ experiences of transitional care to older adults with artificial pacemakers.	Purposive	• Semi-structured interviews; • Discourse of the collective subject methodological framework	Philanthropic hospital	14 female nurses, a mean age of 37 years. Most had >5 years of working experience (*n*=9) and a graduate education level (*n*=13).	This study found that nurses emphasize the importance of prioritizing patient care in the transition from hospital to home for older adults with cardiac pacemakers, highlighting deficiencies in care planning. It identifies factors affecting transitional care, exacerbated by limited knowledge among participants, yet underscores the necessity for skilled care tailored to the specific needs of older adults to maintain their quality of life.
17. Agerholm et al. (2023)/Sweden	To examine health care professionals’ experiences of barriers and facilitators for care coordination for older adults with complex health and social care needs.	Purposive	• Semi-structured interviews; • Thematic analysis	Primary health care center	25 nurses, demographic data unclear.	This study found three overarching themes: (1) methods of communication, (2) the organizational structures in place, and (3) the additional efforts made by staff members.
18. Lindberg et al. (2023)/Sweden	To examine nurses’ experiences of maintaining risks for home-­dwelling older patients.	Purposive	• Semi-structured interview; • Inductive content analysis	Municipal health care center	13 nurses (9 females), age ranged from 30 to 60 years old, had 1–25+ years of RN experience, with 31% having 1–5 years, 38% with 6–10 years, 24% with 11–15 years, 8% with 16–20 years, and 8% with over 25 years of experience.	This study found that registered nurses actively sought to ensure a secure environment for patient care, taking a proactive stance in their roles and endeavoring to anticipate the patient’s needs by staying one step ahead.
19. Winqvist et al. (2023)/Sweden	To examine nurses’ experiences regarding structural conditions of good quality care during transitions from hospital to home health care.	Purposive and theoretical	• Interviews and field notes; • Thematic analysis	Municipal health care center	21 nurses (20 females), aged 26–64 years old, working experiences ranged from 2 to 30 years.	This study found that the structural conditions influencing care transitions can be categorized into three themes: (1) distances and inaccessibility, (2) competence of the actors, and (3) levels of organizational governance.
20. Peeler et al. (2024)/United States	To examine the experiences of patients, family caregivers, and health care professionals in intermediate care units.	Convenience	• Interviews; • Inductive thematic analysis	Intermediate care units of an academic medical center	4 nurses, demographic data unclear.	This study found that five factors are crucial for patient care, including (1) the significance of the relationship between patient and provider, (2) the necessity for transparent and truthful communication, (3) the availability of resources both during hospital stays and upon discharge, (4) the provision of support, training, and education for caregivers, and (5) the implementation of coordinated care and the assurance of follow-up services.

^a^ Studies are listed chronologically.

The four main themes identified across all of the studies included (1) challenges in accessing and delivering health care services, (2) challenges in communications and coordination, (3) need to advance nurses’ roles and recognize their contributions, and (4) importance of providing patient-centered care. The main themes and subthemes are shown in Table 3.

**Table 3:** Identified Themes and Subthemes

Theme	Subtheme
1. Challenges in accessing and delivering health care services	(1) Awareness and access to services (2) Delivering care to aging populations
2. Challenges in communications and coordination	(1) Discontinuities in care transitions (2) Communication and collaboration with other health care providers
3. Need to advance nurses’ roles and recognize their contributions	(1) Desire for greater involvement in care decisions (2) Professional development and career advancement
4. Importance of providing patient-centered care	(1) Respecting patient autonomy (2) Addressing patient-identified concerns (3) Building trust and rapport (4) Understanding the whole person

### Theme 1: Challenges in Accessing and Delivering Health Care Services

The significant barriers to accessing and delivering health care services, particularly for the aging population, are described in this theme. A critical knowledge gap exists not only among patients but also within the health care provider community regarding the spectrum of available services and benefits designed to support older adults. The difficulties are compounded by insufficient dissemination of information and inadequate proactive communication from health care professionals. This theme acknowledges the necessity of bridging this knowledge gap and emphasizes the urgent need to provide comprehensive education on these issues to both health care providers and patients.

#### Subtheme 1.1: Awareness and access to services

Nurses often lack information about the benefits and services available to the aging population, leading to resource underutilization. Nurses face challenges in obtaining necessary information about the health status and care needs of their patients from other health care professionals and institutions. This lack of information coordination may lead to fragmented care and potential gaps in care delivery. For example, a nurse explained their experience with not receiving the necessary information:

Her doctor had never mentioned it. The [doctor and clinic]… has no idea of what the benefits are available out there for people. (Magilvy & Congdon, 2000, p. 339)

#### Subtheme 1.2: Delivering care to aging populations

Nurses encounter systemic and organizational barriers such as limited resources, staffing shortages, and bureaucratic challenges that hinder the provision of care. These barriers may lead to delays in care, reduced access to necessary services, and increased stress for both patients and health care providers. For example, one nurse noted experiencing limited resources and bureaucratic hurdles:

There is only a doctor and a nurse. Sometimes all demands come on the same day and we are a few staff with a few ambulances for patient transfer. (Dolu et al., 2021, p. 876)

Nurses take into account patients’ living environments when delivering care. They observe aspects of the home that may affect patients’ health and well-being and strive to provide care that is tailored to the specific needs of the patient and their home environment. For example, one nurse said:

I look a lot at the environment…how it’s furnished…//…are there pets? Is it clean? Is there alcohol? (Lindberg et al., 2023, p. 3)

Nurses also encounter difficulties accessing medical assessments, managing patients’ psychosocial issues, and navigating complex care systems. They also face issues such as patients’ nonadherence to treatment plans due to financial constraints or living conditions. For example, one nurse said:

I don’t always rule out non-adherent first because there may be a reason. I assess the whole situation… Another thing is their environment. You know, where do they live? Do they have access to get the medicine or the right foods for their diet? (Irani et al., 2020, p. 6)

Providing care in rural areas causes unique challenges to care delivery. These include travel distance, transportation issues, and limited access to services. These challenges require innovative solutions and policy interventions to improve the efficiency and effectiveness of health care delivery in rural settings. For example, one nurse from a rural area reported:

…when it comes to sparsely populated rural areas, as there can be a long distance to the closest neighbor, they still keep an eye on each other, like a small security team of their own. (Winqvist et al., 2023, p. 8)

### Theme 2: Challenges in Communications and Coordination

The difficulties in ensuring effective communication and coordination among health care providers, patients, and their families are addressed in this theme. It includes issues such as medication management, discharge planning, and continuity of care. This theme reflects the complexity of navigating health care systems and the need for clear communication channels to prevent readmissions, medication errors, and other adverse outcomes. It also points to the necessity of establishing robust systems for information sharing and care coordination to improve patient care transitions.

#### Subtheme 2.1: Discontinuities in care transitions

Nurses encounter difficulties in ensuring transitions of care from the hospital to primary care settings are smooth for older patients. There are common delays in communication, leading to other health care providers such as general practitioners (GPs) being unaware of patients’ discharge summaries and updated medication lists, which results in patients having to explain their hospital stay to their GPs. For example, one nurse reported their experience with a discharge issue:

The GP doesn’t have a discharge summary, does not have an updated medication list and the patient turns up at the appointment… So here you’ve got the patient trying to explain to the GP what happened to them in hospital to fill in the picture and tell them something while they are at the appointment. (Mc Namara et al., 2017, p. 295)

Transitions from hospital to home or community-based care are often challenging for nurses. Gaps in communication and coordination between health care professionals result in difficulties in ensuring continuity of care for aging patients. For example, one nurse reported:

I feel like no one is in charge during the transition, leaving the ward and heading home. There is none responsible, or we are poorly informed about who is responsible. (Winqvist et al., 2023, p. 7)

Discharge processes are a critical component of the health care continuum, as they mark the transition from hospital-based care to community-based care. For example, one nurse said:

Discharge guidelines start from admission… I start preparing them from admission. (Santos Oliveira et al., 2022, p. 3)

#### Subtheme 2.2: Communication and collaboration with other health care providers

Nurses encounter communication and collaboration barriers with other health care professionals, especially during care transitions. Lack of common understanding, terminology differences, and economic incentives have been reported to hinder effective communication and collaboration. For example, one nurse said:

Public hospitals do not deal much with patients after discharge… and the carer should take the patient to the hospital. (Dolu et al., 2021, p. 875)

Nurses recognize the importance of interprofessional collaboration in improving care for aging patients. They emphasize the need for better communication and teamwork among different health care providers to ensure continuity of care. For example, one nurse shared their views on interprofessional collaborative relationships:

The primary healthcare gerontology nurse specialist (PHC-GNS) has integrated very well with our team and our service… It’s just great to have a good relationship with someone that’s in primary care… She knows our service, she knows how it works and the doors always open if she’s got a problem or if she needs some direction within secondary care. (A. I. I. King et al., 2018, p. 814)

### Theme 3: Need to Advance Nurses’ Roles and Recognize Their Contributions

The critical role nurses play in patient care and their desire for greater involvement in decision-making processes are highlighted in this theme. Despite their significant contributions, nursing staff often feel undervalued and thus desire and seek recognition for their expertise and dedication. The need for greater acknowledgment of the nursing role in patient care and to empower nurses to have a more active voice in care planning and management are emphasized.

#### Subtheme 3.1: Desire for greater involvement in care decisions

Nurses express a desire to have more involvement in care decisions, reflecting a need for greater recognition of their expertise. Nurses encounter challenges in being fully recognized and valued for their contributions in the care of aging patients. There is often a need for nurses to educate GPs on the scope of their practice and skills. Nurses also express a desire to be more involved in chronic disease management. For example, one nurse said:

I think it’s a constant battle to educate them [GPs] on what we can actually do…. we’re nowhere near respected enough for what knowledge we have and what experience we bring to the role. (McInnes et al., 2017, p. 1963)

#### Subtheme 3.2: Professional development and career advancement

The development of advanced nursing practice roles such as nurse practitioners (NPs) enhances care quality and continuity in several ways. NPs provide comprehensive patient-centered care, serve as a consistent point of contact for patients, reduce health care costs, and help address health care workforce shortages. By leveraging the skills and expertise of NPs, health care systems can improve patient outcomes, increase patient satisfaction, and enhance the overall health care experience. For example, one nurse said:

It would certainly increase the quality of care for our elderly to have this role. (Lindberg et al., 2023, p. 4)

### Theme 4: Importance of Providing Patient-Centered Care

The importance of tailoring health care services to individual patient needs, preferences, and values is highlighted in this theme. It involves actively listening to patients, respecting their autonomy, and prioritizing their personal health goals. Nurses practicing patient-centered care work collaboratively with patients to identify what matters to them most. This approach requires building trust and rapport, which is achieved through consistent and empathetic engagement and communication.

#### Subtheme 4.1: Respecting patient autonomy

Nurses emphasize the importance of respecting patients’ wishes and values, even when these do not align with the nurse’s assessment of needs. For example, one nurse said:

We come from the platform and perspective of, we’re going in there to look at what the client wants, rather than what we want. (Peart et al., 2020, p. 2323)

#### Subtheme 4.2: Addressing patient-identified concerns

Care is most effective when nurses address issues of concern to patients, which may differ from the clinical priorities. This approach, often referred to as patient-centered care, recognizes patients as unique individuals with their own values, beliefs, and preferences that can significantly impact their health and well-being.

When nurses prioritize patient concerns, they are better able to understand their perspectives, identify their unique needs, and develop individualized care plans tailored to their specific situation. This approach may lead to improved patient outcomes, better patient satisfaction, and more patient involvement in their care. For example, one nurse noted:

I believe it is an underdiagnosed group … They don’t come and ask for help. Instead, they believe [that] this is how it is; it’s the trajectory of life, but they can actually get help. (Borglin et al., 2019, p. 3)

#### Subtheme 4.3: Building trust and rapport

Establishing a trusting relationship between health care providers and patients is essential for patient engagement and care-plan compliance. Trust is the foundation of any successful health care relationship, as it enables patients to feel comfortable sharing their health concerns, fears, and personal information with their health care providers. When patients trust their health care providers, they are more likely to adhere to treatment plans, follow medical advice, and actively participate in their care. For example, one nurse said:

You now spent a lot of time on patient education on chronic conditions, what their medications are, things that they can do… Those sorts of things are all there with every patient that I see, but the ones that really matter to the patients are the ones that change their quality of life. (A. I. I. King et al., 2018, p. 813)

#### Subtheme 4.4: Understanding the whole person

Nurses strive to see beyond the medical condition and understand the life context of their patients, which includes their living environment and social situation. This theme reflects the comprehensive assessment and consideration of all factors affecting a patient’s health and well-being. A holistic approach goes beyond addressing immediate medical issues to include understanding the patient’s broader life context, including their living environment, social support network, and psychological state. By considering the whole person, nurses can develop more effective and personalized care strategies that address the root causes of health issues and promote overall well-being. For example, one nurse reported:

You have to work with them to get a good understanding of… what they value in life, their memory, their family, their home. (Peart et al., 2020, p. 2323)

## Discussion

This was the first study to undertake a meta-synthesis of existing works examining the fragmented care experiences of nurses in the context of older adult patients. Nurses reported difficulties in accessing and delivering health care services to aging populations, highlighting barriers to care. Prevalent problems related to communication and coordination indicate a need for improved information sharing and collaboration between health care providers. The role and recognition of nursing staff were found to be significant, with nurses reporting a need for greater support as well as recognition of their contributions to care. In addition, the significance of patient-centered care was emphasized, with nurses recognizing the importance of providing individualized care that addresses the unique needs and preferences of older adult patients.

Accessibility and acceptability have previously been identified as two significant factors that pose challenges for older adults in accessing health care (Mohd Rosnu et al., 2022). This meta-synthesis revealed a similar theme, highlighting the barriers that older adults face in accessing and receiving appropriate care. Barriers, including constraints on resources, staffing shortages, and bureaucratic hurdles, impede the effective delivery of health care. Although nurses play a critical role in identifying and addressing these barriers, they face challenges from inadequate resources, a lack of training, and high workloads. To address these challenges, health care systems should prioritize the unmet needs of older adults and ensure nurses have the necessary resources and support to provide high-quality care.

The significance of effective communication and coordination among health care professionals in delivering continuous care to older adults has been well-established (Sheehan et al., 2021). Inadequate communication and coordination may result in disjointed care, increased hospital and emergency department visits, unnecessary diagnostic tests, and higher medical expenses (B. J. King et al., 2013; Tiwary et al., 2019). Furthermore, positive correlations between relational coordination and, respectively, job satisfaction, work engagement, reduced burnout and turnover, and reciprocal learning among health care professionals have been identified (House et al., 2022). However, there is currently no universally accepted standard for communication. Sheehan et al. (2021) identified four facilitators of effective communication between hospital allied health and primary care practitioners, including multidisciplinary care plans, patient and caregiver involvement, health information technology, and assigning a designated individual for follow-up/care management. Scholars from diverse fields have recommended that leaders and organizations should prioritize fostering a collaborative culture, socializing members to embrace collaboration, promoting care coordination, and comprehending health care as a complex adaptive system (Bolton et al., 2021; Persson et al., 2022). Moreover, incorporating collaborative skills training across professions may further enhance care coordination within educational programming. To tackle the abovementioned challenges, health care systems should prioritize interprofessional collaboration and communication to ensure nurses are integral to care planning and decision-making processes. Furthermore, the vital role nurses play in communicating with patients may be leveraged to further enhance nursing performance, increase patient involvement in health matters, decrease misunderstandings between nurses and patients, and raise awareness of preventive health care (Senek et al., 2020). Nevertheless, negative perceptions and misconceptions can impede the development of effective nurse–patient relationships.

The findings of this study highlight the coordinator role that nurses often undertake in multidisciplinary teams and their appreciation of opportunities to collaborate with other health care professionals, which aligns with previous research in long-term health care contexts (Nasu et al., 2020). Although physicians generally recognized their dependence on nurses and appreciate the geriatric and palliative care competencies of nurses (Lee et al., 2014), nurses often face barriers to communicating with other health care professionals and to conveying thoughts that their work and potential contributions are undervalued (Sekse et al., 2018). To promote more effective interdisciplinary communication and teamwork, it is necessary to enhance knowledge of each profession’s roles and functions through targeted education and training.

The findings of this synthesis revisit the criticality of delivering patient-centered care tailored to the distinct needs and predilections of older adults, which aligns with the findings of prior studies (Moore et al., 2017). Four key components of patient-centered care from the perspective of nurses were identified in this study: honoring patient autonomy, focusing on concerns perceived as important by the patient, building up trust and rapport, and appreciating the patient’s holistic context. These components echo the findings of prior research (Entwistle et al., 2010; Narayan, 2022). Incorporating these components into daily nursing practice not only enhances patient satisfaction but also contributes to improved health outcomes by fostering a supportive environment that encourages patient engagement in their care. This alignment with patient values and preferences is essential for delivering high-quality health care. To optimize the care available to older adult patients, health care systems should ensure nursing staff are equipped with the training and resources necessary to offer tailored care that meets the specific needs and preferences of the geriatric population.

### Limitations

This meta-synthesis has several limitations that may impact the generalizability and validity of findings. First, the inclusion criteria may have introduced selection bias through the omission of relevant studies with additional insights into this issue. Although the search strategy used was comprehensive, some scholars have delineated distinctions among concepts such as care fragmentation, care continuity, and care coordination (Kern et al., 2024). Second, the exclusion of non-English language studies may have limited the cultural and linguistic diversity of the included studies, thus potentially limiting the transferability of the findings. Furthermore, while the synthesis included studies involving nursing staff, it did not explicitly focus on nurses with specialized roles such as nurse practitioners, community nurses, and nursing managers. Finally, health care systems demonstrate significant diversity worldwide and are shaped by factors such as economic conditions, political structures, cultural norms, and technological advancements. For instance, the significant differences in health care policies and regulations from country to country influence patient rights, health care access, professional scope of practice, and other aspects (Jutz, 2020). Given this variability, the findings of any specific research synthesis or study on fragmented care may not be universally transferable across all health care systems. Notably, most of the reviewed studies investigated nurse experiences in Western countries, underscoring the need for research from underrepresented regions such as Asia. The absence of studies from non-Western nations does not necessarily reflect the prevalence of fragmented care in those contexts but rather highlights the need for increased scholarly attention to this issue globally.

### Conclusions

This meta-synthesis provides key insights into the fragmented care experiences of nurses in the context of older adult health care. The significant challenges to accessing appropriate services under fragmented care scenarios highlight the need for effective communication and coordination among health care providers to ensure quality care. Nurses play a crucial role in addressing these issues and require adequate support and recognition to fulfill their responsibilities effectively. In addition, prioritizing individualized care that addresses the unique needs and preferences of older adults will be essential to improving the overall health care experience of this patient group. To address these challenges, health care systems should prioritize the needs of older adults and implement strategies tailored to the unique context of each system. This may involve integrating care services, improving communication among providers, and leveraging electronic health records. However, the implementation and effectiveness of these strategies will be influenced by the specific characteristics and constraints of the local health care system. Therefore, it is crucial to consider the unique context and setting of each system when applying these research findings to improve the quality of health care provided to older adults.
